# The *Deepwater Horizon* Oil Spill and Physical Health among Adult Women in Southern Louisiana: The Women and Their Children’s Health (WaTCH) Study

**DOI:** 10.1289/ehp.1510348

**Published:** 2016-01-22

**Authors:** Lauren C. Peres, Edward Trapido, Ariane L. Rung, Daniel J. Harrington, Evrim Oral, Zhide Fang, Elizabeth Fontham, Edward S. Peters

**Affiliations:** 1 Department of Epidemiology,; 2 Department of Environmental and Occupational Health Sciences, and; 3 Department of Biostatistics, Louisiana State University Health Sciences Center School of Public Health, New Orleans, Louisiana, USA

## Abstract

**Background:**

The Deepwater Horizon Oil Spill (DHOS) is the largest oil spill in U.S. history, negatively impacting Gulf Coast residents and the surrounding ecosystem. To date, no studies have been published concerning physical health outcomes associated with the DHOS in the general community.

**Objectives:**

We characterized individual DHOS exposure using survey data and examined the association between DHOS exposure and physical health.

**Methods:**

Baseline data from 2,126 adult women residing in southern Louisiana and enrolled in the Women and Their Children’s Health study were analyzed. Exploratory factor analysis was used to characterize DHOS exposure. Odds ratios and 95% confidence intervals for the associations between DHOS exposure and physical health symptoms were estimated using multivariate logistic regression.

**Results:**

A two-factor solution was identified as the best fit for DHOS exposure: physical–environmental exposure and economic exposure. High physical–environmental exposure was significantly associated with all of the physical health symptoms, with the strongest associations for burning in nose, throat, or lungs (OR = 4.73; 95% CI: 3.10, 7.22), sore throat (OR = 4.66; 95% CI: 2.89, 7.51), dizziness (OR = 4.21; 95% CI: 2.69, 6.58), and wheezing (OR = 4.20; 95% CI: 2.86, 6.17). Women who had high-economic exposure were significantly more likely to report wheezing (OR = 1.92; 95% CI: 1.32, 2.79); headaches (OR = 1.81; 95% CI: 1.41, 2.58); watery, burning, itchy eyes (OR = 1.61; 95% CI: 1.20, 2.16); and stuffy, itchy, runny nose (OR = 1.56; 95% CI: 1.16, 2.08).

**Conclusions:**

Among southern Louisiana women, both physical–environmental and economic exposure to the DHOS were associated with an increase in self-reported physical health outcomes. Additional longitudinal studies of this unique cohort are needed to elucidate the impact of the DHOS on short- and long-term human health.

**Citation:**

Peres LC, Trapido E, Rung AL, Harrington DJ, Oral E, Fang Z, Fontham E, Peters ES. 2016. The Deepwater Horizon Oil Spill and physical health among adult women in southern Louisiana: the Women and Their Children’s Health (WaTCH) study. Environ Health Perspect 124:1208–1213; http://dx.doi.org/10.1289/ehp.1510348

## Introduction

On 20 April 2010, the British Petroleum (BP) *Deepwater Horizon* drilling rig exploded off the coast of Louisiana, killing 11 workers (National Commission on the BP *Deepwater Horizon* Oil Spill and Offshore Drilling 2011). Subsequently, 200 million gallons of crude oil spilled into the Gulf of Mexico over the next 3 months, making the *Deepwater Horizon* Oil Spill (DHOS) the largest oil spill in United States history (Goldstein et al. 2011). BP used controlled burns and 1.84 million gallons of dispersant chemicals to break up the crude oil, and employed several thousand workers and volunteers to aid in the clean-up activities (Biello 2010; National Commission on the BP *Deepwater Horizon* Oil Spill and Offshore Drilling 2011). Even with these considerable efforts, the Gulf Coast was severely affected by the DHOS, with damages to the rich ecosystem, impacting both the tourism and fishing industries, and threats to the physical and mental well-being of residents, clean-up workers, and volunteers (National Commission on the BP *Deepwater Horizon* Oil Spill and Offshore Drilling 2011).

Several studies have described the negative impact of oil spills and other man-made or natural disasters on human health (Aguilera et al. 2010; Giorgadze et al. 2011; Goldmann and Galea 2014; Norris et al. 2002; Rung et al. 2015). Crude oil and dispersants contain many components that are toxic to humans, such as aromatic/aliphatic hydrocarbons, hydrogen sulfide gas, and sulfonic acid (Solomon and Janssen 2010). Physical contact with, inhalation, or ingestion of these toxic components may result in a variety of adverse physiologic effects in the immediate aftermath of an oil spill, including respiratory irritations, central nervous system depression, and high doses of exposure to some components could also increase the risk of cancer (Solomon and Janssen 2010). It has been proposed that social and economic disruption resulting from the DHOS might also have an indirect effect on the health of exposed populations (Goldstein et al. 2011). Many Gulf Coast residents rely on the local fishing and tourism industries for their livelihood, which were severely affected by the DHOS and its damage to the surrounding ecosystem (National Commission on the BP *Deepwater Horizon* Oil Spill and Offshore Drilling 2011). Financial burden, in general, often negatively impacts the mental and psychosocial well-being of an entire household (Pearlin et al. 2005; Tosevski and Milovancevic 2006) and may manifest as physical health problems (Cohen et al. 2007). A review article conducted by Aguilera et al. (2010) examined several epidemiologic studies that explored the resultant health effects after exposure to oil spills, finding adverse neurological and mental health outcomes as well as respiratory and dermal irritations for those exposed to oil spills. The DHOS has often been compared to the second largest oil spill in the U.S., the *Exxon Valdez* oil spill, which released thousands of barrels of oil into Prince William Sound, Alaska, and has been negatively associated with the health of surrounding communities (Nelson et al. 2014). Several studies have examined health outcomes following the *Exxon Valdez* oil spill, noting that individuals exposed to the spill reported higher rates of economic disruption and an increase in adverse mental health outcomes, including stress, anxiety, and depression (Palinkas et al. 1993a, 1993b). To date, only a few studies have examined the health effects of the DHOS. D’Andrea and Reddy (2013) examined the physical health of clean-up workers following the DHOS and reported a higher frequency of headaches, shortness of breath, skin rash, and cough among the workers in comparison to unexposed Gulf Coast Louisiana residents. Another study examined mental health effects of individuals residing in southern Louisiana after the DHOS, noting that disruption of the family and work environment by the DHOS was associated with negative mental health outcomes, especially anxiety and depression (Osofsky et al. 2011).

Although there have been a number of oil spills around the world, with several studies examining their impact on human health, a consistent, reliable, and valid measurement of an individual’s oil spill exposure has not been established or utilized throughout the disaster literature. Defining what constitutes oil spill exposure is a crucial step in studying and understanding the impact oil spills can have on human health and well-being. In addition, traditional epidemiologic studies examining health related effects of oil spills have focused mainly on clean-up workers (Aguilera et al. 2010). Although clean-up workers are the most likely persons to be directly exposed to the crude oil, massive oil spills, such as the DHOS, have a far-reaching effect on surrounding communities and their residents. To date, there has been a paucity of research on community members affected by a substantial oil spill; therefore, we focused on studying the health of adult women residing in the most heavily affected areas of Louisiana. We sought to use survey data to characterize individual DHOS exposure using exploratory factor analysis and to examine the association between DHOS exposure and physical health immediately following the DHOS.

## Methods

### Study Population

Baseline data were obtained from a cohort of adult women enrolled in the Women and Their Children’s Health (WaTCH) study. The WaTCH study is an ongoing, prospective cohort study that aims to examine the short- and long-term physical, mental, and community health effects resulting from the DHOS among women and children residing in Southeast Louisiana. Eligibility for the study required women to be 18–80 years of age and a resident of one of the seven most heavily affected parishes (county equivalent) in Louisiana (Orleans, St. Bernard, Jefferson, Plaquemines, Lafourche, Terrebonne, and St. Mary) at the time of the oil rig explosion, 20 April 2010. Identification of adult women occurred through address-based sampling. Marketing Systems Group (Horsham, PA, USA), which uses the U.S. Postal Service’s Computerized Delivery Sequence File that covers 100% of all U.S. households, provided lists of individual and household addresses. Women were also recruited through referrals of friends and neighbors, as well as volunteers. Identified women were first sent a letter introducing the study; following the letter, each woman was contacted by telephone to complete an extensive computer-assisted survey consisting of questions on demographics, physical and emotional health, lifestyle behaviors, environmental oil exposure, and occupational history. All WaTCH data were collected and managed using Research Electronic Data Capture (REDCap) tools hosted at the Epidemiology Data Center at the Louisiana State University Health Sciences Center. REDCap is a secure, web-based application designed to support data capture for research studies; it provides *a*) an intuitive interface for validated data entry, *b*) audit trails for tracking data manipulation and export procedures, *c*) automated export procedures for seamless data downloads to common statistical packages, and *d*) procedures for importing data from external sources (Harris et al. 2009). The WaTCH study was reviewed and approved by the Louisiana State University Health Sciences Center Institutional Review Board, including a waiver of documentation of informed consent. Verbal informed consent was provided by the participants over the telephone.

Between July 2012 and August 2014, calls were made to 42,649 telephone numbers in an attempt to reach potentially eligible women for participation in the WaTCH study. Of the 42,649 telephone numbers attempted, 16,732 numbers were deemed ineligible (disconnected numbers, no woman at the telephone number, businesses, etc.), 67 numbers were of known eligibility but refused or were noncontacts, and 22,998 numbers were of unknown eligibility (never picked up, hang-ups before determination of eligibility). The final sample consisted of 2,852 women that completed the baseline telephone questionnaire. In an effort to standardize response rates across survey literature, the response rate was calculated using the American Association of Public Opinion Research’s response rate calculator (AAPOR 2015). The response rate was calculated by dividing the number of complete interviews by the number of eligible units, multiplied by an estimated proportion of unknown eligibility units that are actually eligible (14.9% for the WaTCH study). To determine this estimated proportion, the number of known eligible units (completed interviews and eligible women that refused or were noncontacts) was divided by the number of all units in the sample for which a definitive determination of status was obtained (completed interviews, eligible women that refused or were noncontacts, and ineligible women). The estimated overall response rate was 45%.

### Physical Health Symptoms

Study participants were asked to report how often thirteen physical health symptoms occurred between 20 April and 25 December 2010, the 8-month time period immediately following the DHOS. The following physical health symptoms were assessed: cough; wheezing or tightness in chest; shortness of breath; watery, burning, or itchy eyes; stuffy, itchy, or runny nose; burning in nose, throat, or lungs; skin rash, sore, or blister that lasted more than 3 days; dizziness; severe headaches or migraines; nausea; blurred or distorted vision; excessive fatigue or extreme tiredness; and sore throat. Response choices were recorded on a 5-point Likert scale, and included “All of the time,” “Most of the time,” “Sometimes,” “Rarely,” or “Never”. WaTCH participants who responded “All of the time” or “Most of the time” were categorized as having the symptom of interest.

### Characterizing DHOS Exposure

To characterize exposure to the DHOS, the WaTCH study utilized six oil spill exposure questions that Palinkas et al. (1993b) developed for the 1989 *Exxon Valdez* oil spill and added further questions on the financial impact of the DHOS and the participant’s ability to smell the oil. Table 1 presents the WaTCH study questions used in the survey to assess potential exposures to the DHOS. A latent variable approach, exploratory factor analysis (EFA), was used to quantify exposure to the DHOS. EFA is a variable-centered method that extracts a number of factors from the data to account for the correlation patterns among the variables, without using an *a priori* factor structure of the measured outcomes (Fabrigar et al. 1999). The EFA was conducted using M*plus*, version 7 (Muthen & Muthen, Los Angeles, CA, USA), and was restricted to only those women providing responses to all nine of the oil spill indicators (*n* = 2,584). Weighted least squares mean and variance adjusted estimation was used to explore the factor structure of the nine DHOS indicators, which is the preferred method to handle categorical data (Schmitt 2011). An oblique rotation, GEOMIN, was used to allow factors to be correlated with one another. Factor solutions were generated for a possibility of 1–4 factors, and the scree plot, fit indices (Comparative Fit Index, Tucker Lewis Index, and the root mean square error of approximation), and the interpretability of each factor were evaluated to determine the best model fit and factor solution (Costello and Osborne 2005; Ford et al. 1986).

**Table 1:** DHOS exposure items asked of WaTCH participants in the telephone survey.

DHOS exposure items	Answer choices
1. Did you work on any of the oil spill cleanup activities?	Yes/no
2. Are there any other ways that you came into physical contact with the oil from the spill or cleanup activities?	Yes/no
3. Did you have any property that was lost or damaged because of the oil spill or cleanup?	Yes/no
4. Did the oil spill cause any physical damage to the areas where you or other household members fish commercially?	Yes/no
5. Has the oil spill directly affected the recreational hunting, fishing, or other activities of any members of this household?	Yes/no
6. Did you or anyone in your household lose any income due to disruption of employment or closing a business because of the oil spill?	Yes/no
7. Compared to other residents in your community, were you:^*a*^	Hit harder, affected about the same, or affected less by the oil spill
8. How would you rate the influence of the oil spill on your household’s current financial situation?^*b*^	Very negative, somewhat negative, somewhat positive, very positive, or no influence
9. After the oil spill, could you smell the oil?^*c*^	Yes/no
9a. If yes, how strong was the smell?	Not strong, a little strong, moderately strong, quite strong, or extremely strong
9b. If yes, how often could you smell it?	None, a little of the time, some of the time, most of the time, or all of the time
^***a***^Categorized as hit harder by the oil spill versus affected about the same or affected less by the oil spill. ^***b***^Categorized as very or somewhat negative influence versus no influence, very or somewhat positive influence. ^***c***^A summary score was created to indicate strength and frequency of smell using the three smell exposure questions. If a participant reported smelling the oil, the Likert responses to the follow-up questions on strength and frequency of smell were summed to create a summary score. If a participant did not report smelling the oil, their summary score was a value of 0. The score ranged from 0 to 9 and was dichotomized into two groups based on the median: none to low smell (0–4) and medium to high smell (5–9).	

### Statistical Analysis

The remaining statistical analyses were conducted using SAS (version 9.3; SAS Institute Inc., Cary, NC, USA) and were restricted to include only WaTCH participants for whom data on oil spill indicators, physical health symptoms, and covariates were available (*n* = 2,126). Descriptive statistics were calculated for demographic characteristics of WaTCH participants and multivariate logistic regression was performed to estimate odds ratios (OR) and 95% confidence intervals (CI) for the relationships between exposure to the oil spill and physical health symptoms. The following variables were considered *a priori* confounders and were adjusted for in all models: age (years; continuous), household income in the year prior to the DHOS (< $20,000, $20,001–$40,000, $40,001–$60,000, > $60,000), smoking status at the time of interview (never, former, and current smoker), and race (white, black, other). The category “other race” included women who identified as Asian/Pacific Islander, American Indian, other race, or multiracial. We also evaluated whether the time of the interview (number of years from the date of the oil rig explosion to the date of the interview) was a potential confounder in the relationship between oil spill exposure and physical health. The time of the interview did not appreciably change the parameter estimates (< 10% change in the ORs) and was not included as a confounder in subsequent analyses (data not shown). The effect estimates for one factor were also adjusted for the other factor of the best fitting EFA model solution. The Bonferroni correction method was used to adjust for multiple comparisons. The a-level of 0.05 was divided by the number of tests (*n* = 13) to arrive at a new threshold of statistical significance, *p* < 0.0038.

## Results

### Study Population

Table 2 outlines the characteristics of the study population. The mean age of adult women was 45.1 ± 11.7 years, and the majority of the study population was white (58.3%), with 35.6% black and 6.1% other race. About half of the women reported an annual household income of < $40,000 (45.8%), and 20.1% reported currently smoking. A small percentage of women worked on the DHOS clean-up activities (2.2%), had property that was lost or damaged due to the DHOS (2.5%), and reported physical damage to the commercial fishing areas used by members of their household (7.0%). Participants reported the highest impact on their financial situation, with 38.2% reporting a negative or somewhat negative impact on household finances and 25.5% reporting losing income due to disruption of employment or the closing of a business.

**Table 2:** Characteristics of WaTCH participants (*n* = 2,126).

Characteristics	*n* (%) or Mean ± SD
Age (years)	45.1 ± 11.7
Race
White	1,240 (58.3)
Black	757 (35.6)
Other^*a*^	129 (6.1)
Household income
$0–$20,000	523 (24.6)
$20,001–$40,000	451 (21.2)
$40,001–$60,000	338 (15.9)
$60,001+	814 (38.3)
Smoking status
Never smoker	1,366 (64.3)
Former smoker	332 (15.6)
Current smoker	428 (20.1)
Oil spill exposure indicators
Worked on clean-up activities	46 (2.2)
Came into physical contact with oil in other ways	458 (21.5)
Property lost or damaged due to the DHOS	52 (2.5)
Physical damage to areas where you fish commercially	148 (7.0)
DHOS affected recreational activities of anyone in household	714 (33.6)
Lost income at a business due to the DHOS	543 (25.5)
Hit harder by the DHOS than others in community	127 (6.0)
Negative or somewhat negative impact on household finances	811 (38.2)
Medium-high strength/frequency of the oil smell	561 (26.4)
SD, standard deviation. ^***a***^Other race includes women who identified as Asian/Pacific Islander, American Indian, other, or multiracial.	

### Factor Analysis Solution for DHOS Exposure

After evaluating the fit indices and interpretability of each factor solution, a two-factor solution was identified as the best fit for the data. Using a threshold of 0.30, all of the indicators significantly loaded on one of the two factors. The rotated factor loadings for the two-factor solution are provided in Table 3. Based on the indicators that loaded high on each factor, Factor 1 was labeled as “Physical–Environmental Exposure to the DHOS” and Factor 2 was labeled as “Economic Exposure to the DHOS.” The binary responses to the questions included in each factor were then summed to create an exposure score. The range of possible scores for physical–environmental exposure was 0–6, and for economic exposure, 0–3. Exposure scores were then categorized into three groups: unexposed, low exposure, and high exposure. An exposure score of zero was defined as unexposed and the cut points for low and high exposure were determined by the median exposure score (a score of 1 for both physical–environmental and economic exposure).

**Table 3:** Rotated factor loadings of the DHOS indicators for the two-factor solution.

DHOS Indicators	Rotated factor loadings	
	Factor 1: Physical–environmental exposure to the DHOS	Factor 2: Economic exposure to the DHOS
Worked on clean-up activities	0.449	–0.045
Came into physical contact with oil in other ways	0.723	–0.144
Property lost or damaged due to the DHOS	0.606	0.171
Physical damage to areas where you fish commercially	0.569	0.201
DHOS affected recreational activities of anyone in household	0.735	0.002
Lost income at a business due to the DHOS	–0.010	0.876
Hit harder by the DHOS than others in community	0.170	0.553
Negative or somewhat negative impact on household finances	0.110	0.694
Medium-high strength/frequency of the oil smell	0.443	0.043
Note: Shaded areas represent the indicators included in each factor.		

### Logistic Regression Analyses

The estimated odds ratios and 95% confidence intervals for each physical health symptom are presented in Table 4 (physical–environmental exposure to the DHOS) and Table 5 (economic exposure to the DHOS). In comparison to no physical–environmental exposure to the DHOS, low physical–environmental exposure was significantly associated (Bonferroni-corrected *p* < 0.0038) with self-report of the following symptoms being present “All of the time” or “Most of the time” in the 8-month time period following the DHOS: wheezing; watery, burning, itchy eyes; stuffy, itchy, runny nose; burning in nose, throat, or lungs; dizziness; fatigue; and sore throat. In general, a dose–response relationship was observed between physical–environmental exposure to the DHOS and all of the physical health symptoms, where the magnitude of the estimated association increased as the level of exposure increased from low to high. All of the physical health symptoms were significantly associated with high physical–environmental exposure to the DHOS. Of these relationships, the symptoms with the strongest associations were burning in nose, throat or lungs (OR = 4.73; 95% CI: 3.10, 7.22); sore throat (OR = 4.66; 95% CI: 2.89, 7.51); dizziness (OR = 4.21; 95% CI: 2.69, 6.58); and wheezing (OR = 4.20; 95% CI: 2.86, 6.17). No significant associations were observed between any of the physical health symptoms and low economic exposure to the DHOS; however, high economic exposure to the DHOS was associated with a few of the physical health symptoms. Compared to women who reported no economic exposure to the DHOS, women with high economic exposure were more likely to report wheezing (OR = 1.92; 95% CI: 1.32, 2.79); headaches (OR = 1.81; 95% CI: 1.41, 2.58); watery, burning, itchy eyes (OR = 1.61; 95% CI: 1.20, 2.16); and stuffy, itchy, runny nose (OR = 1.56; 95% CI: 1.16, 2.08) all or most of the time.

**Table 4:** Estimated adjusted odds ratios and 95% confidence intervals for physical health symptoms by physical–environmental exposure to the DHOS (*n* = 2,126).

Physical health symptoms^*a*^	Physical–environmental exposure to the DHOS^*b*^				
	None (*n* = 979)	Low (*n* = 592)		High (*n* = 555)	
	*n*	*n*	aOR (95% CI)	*n*	aOR (95% CI)
Cough	106	84	1.35 (0.98, 1.86)	103	3.43 (2.54, 4.64)*
Wheezing	49	61	2.10 (1.40, 3.14)*	74	4.20 (2.86, 6.17)*
Shortness of breath	66	64	1.69 (1.16, 2.46)	71	3.37 (2.35, 4.83)*
Watery, burning, itchy eyes	111	118	1.92 (1.43, 2.57)*	114	3.07 (2.30, 4.10)*
Stuffy, itchy, runny nose	116	114	1.72 (1.29, 2.29)*	117	2.90 (2.18, 3.85)*
Burning in nose, throat, lungs	37	57	2.68 (1.73, 4.16)*	63	4.73 (3.10, 7.22)*
Skin rash	28	31	1.69 (0.99, 2.88)	38	3.62 (2.22, 5.89)*
Dizziness	36	44	2.16 (1.35, 3.44)*	44	4.21 (2.69, 6.58)*
Headaches	119	96	1.40 (1.03, 1.90)	120	2.76 (2.06, 3.72)*
Nausea	41	45	1.84 (1.17, 2.88)	53	3.57 (2.34, 5.45)*
Blurry or distorted vision	48	48	1.70 (1.10, 2.63)	34	2.33 (1.50, 3.62)*
Fatigue	156	137	1.56 (1.19, 2.03)*	127	2.57 (1.97, 3.35)*
Sore throat	28	49	3.02 (1.85, 4.91)*	53	4.66 (2.89, 7.51)*
Note: aOR, adjusted odds ratio; CI: confidence interval. ORs adjusted for age, race, income, smoking status, and economic exposure to the DHOS. ^***a***^Symptoms reported “All of the time” or “Most of the time” in the 8-month time period directly following the DHOS, 20 April–25 December 2010. ^***b***^Referent group is no physical–environmental exposure to the DHOS. Low physical–environmental exposure to the DHOS is defined as a score of 1 and high is defined as a score of > 1. *Significant at the Bonferroni adjusted a level, *p* < 0.0038.					

**Table 5:** Estimated adjusted odds ratios and 95% confidence intervals for physical health symptoms by economic exposure to the DHOS (*n *= 2,126).

Physical health symptoms^*a*^	Economic exposure to the DHOS^*b*^				
	None (*n* = 1,160)	Low (*n* = 531)		High (*n* = 435)	
	*n*	*n*	aOR (95% CI)	*n*	aOR (95% CI)
Cough	143	107	1.23 (0.91, 1.66)	103	1.45 (1.06, 1.99)
Wheezing	76	70	1.43 (0.99, 2.07)	74	1.92 (1.32, 2.79)*
Shortness of breath	96	71	1.16 (0.82, 1.65)	71	1.56 (1.08, 2.24)
Watery, burning, itchy eyes	159	120	1.32 (1.00, 1.74)	114	1.61 (1.20, 2.16)*
Stuffy, itchy, runny nose	165	116	1.26 (0.96, 1.67)	117	1.56 (1.16, 2.08)*
Burning in nose, throat, lungs	69	56	1.18 (0.80, 1.74)	63	1.68 (1.14, 2.49)
Skin rash	41	42	1.66 (1.04, 2.65)	38	1.69 (1.04, 2.75)
Dizziness	61	49	1.15 (0.75, 1.74)	44	1.30 (0.84, 2.02)
Headaches	143	112	1.45 (1.08, 1.84)	120	1.81 (1.41, 2.58)*
Nausea	63	51	1.25 (0.83, 1.88)	53	1.48 (0.97, 2.25)
Blurry or distorted vision	63	55	1.36 (0.90, 2.04)	34	1.19 (0.74, 1.91)
Fatigue	211	144	1.28 (0.99, 1.65)	127	1.33 (1.01, 1.76)
Sore throat	53	45	1.28 (0.83, 1.97)	53	1.78 (1.16, 2.73)
Note: aOR, adjusted odds ratio; CI, confidence interval. ORs adjusted for age, race, income, smoking status, and physical–environmental exposure to the DHOS. ^***a***^Symptoms reported “All of the time” or “Most of the time” in the 8 month time period directly following the DHOS, 20 April–25 December 2010. ^***b***^Referent group is no economic exposure to the DHOS. Low-economic exposure to the DHOS is defined as a score of 1 and high is defined as a score of > 1. *Significant at the Bonferroni adjusted a level, *p* < 0.0038.					

## Discussion

In this study, exposure to the DHOS was estimated using exploratory factor analysis, where delineations between physical and economic DHOS exposures were observed. Compared to women who had no physical or environmental exposure to the DHOS, women exposed to the oil spill had significantly higher odds of several physical health symptoms following the DHOS, with the strongest associations estimated for burning in nose, throat, or lungs; sore throat; dizziness; and wheezing. Overall, our findings suggest that both direct exposure to the DHOS and indirect effects of the spill had an impact on the physical health of adult women residing in southeastern Louisiana.

A link between oil spill exposure and acute health outcomes has been consistently reported in the literature, with respiratory and neurologic symptoms reported more frequently (Aguilera et al. 2010). Studies examining the 1996 *Sea Empress* oil spill in Wales and the 1993 grounding of the *Braer* tanker in Scotland reported an increase in eye and throat irritations and headaches among exposed individuals (Campbell et al. 1993; Lyons et al. 1999). To date, two studies have examined the physical health effects of the DHOS, reporting a higher frequency of respiratory illness, headaches, skin rash, and cough among DHOS clean-up workers (D’Andrea and Reddy 2013; King and Gibbins 2011). Similarly, women with high physical–environmental DHOS exposure had an increased odds of reporting all physical health symptoms under investigation. Without pre-spill data on these health outcomes, it is not possible to distinguish whether the findings of this study are causal relationships or if they reflect other environmental exposures or symptoms of common illnesses. In the future, collection of detailed exposure and outcome information at multiple time points will be important in elucidating the effects of disasters on short- and long-term human health.

Some physical health symptoms (wheezing; watery, burning, itchy eyes; stuffy, itchy, runny nose; and headaches) were also associated with high economic exposure to the DHOS. These findings might be a consequence of increased stress levels resulting from the economic burden of the DHOS on the households of many Gulf Coast residents (Cohen et al. 2007). Psychosocial stress has been linked to a host of health consequences, including pain (such as headaches, abdominal pain, etc.) and fatigue (Chrousos 2009). Stress can also impact the regulation of immune and inflammatory processes, increasing an individual’s vulnerability to allergies and respiratory illnesses (Dave et al. 2011; Segerstrom and Miller 2004). In addition, these stressors may increase an individual’s susceptibility to the causative agents of different health outcomes, increasing disease burden (House et al. 1979). The findings from the present study suggest that more attention should be focused on the impact of DHOS-related stressors on not only mental well-being, but also on physical health.

There are considerable methodological challenges in studying the human health effects of disasters due to the lack of pre-disaster data; therefore, studies typically rely on cross-sectional data, as is the case in the present study. With the use of cross-sectional data, temporal or causal relationships cannot be established, and caution should be taken when discussing the implications of results. An additional limitation may arise due to the utilization of landline telephone numbers to recruit WaTCH participants. With an increasing number of younger and low-income adults reporting cell phone usage without a landline (Blumberg and Luke 2009), non-coverage bias may be present in the WaTCH study. Another limitation is that the data were collected through self-report, which may be subject to additional biases, specifically recall and information biases. Adult women may have had impaired recall abilities, especially for the oil spill exposure questions, since WaTCH questionnaire data were collected anywhere from 2.5 to 4.5 years after the oil spill. In addition, measuring oil spill exposure is difficult due to a lack of validated questionnaires measuring oil spill exposure as well as no biomarker of actual exposure. However, this study utilized all available oil spill exposure information collected in the WaTCH study to inform the most appropriate measure of oil spill exposure from the survey data. The challenges of measuring oil spill exposure highlight the importance of developing the infrastructure to rapidly assess exposure post-disasters. Despite these limitations, the present study capitalized on the rich data source of the WaTCH study and its relatively large population of southern Louisiana women. This is the first study to examine the impact of the DHOS among female residents of the Gulf Coast area, and not only on the volunteers and workers involved in the clean-up efforts. Subsequent waves of surveys of this cohort are critical to answering the primary question of the long-term effects of oil spills.

## Conclusion

Overall, this study suggests that physical health was negatively affected by the DHOS, through both direct physical–environmental exposure and indirect economic exposure. It is clear that the DHOS had a diverse impact on the Gulf Coast and its residents, which may continue to persist throughout the population, leaving behind lasting effects. To date, the long-term effects of an oil spill on the health of a community are still unknown. The WaTCH cohort was established to fill this gap in knowledge, and required working with the communities, gathering their input and garnering their support for the study. This unique cohort needs to be followed over time in order to fully elucidate the impact of the DHOS on human health. A thorough assessment of the health and behavior changes following disasters is crucial in determining the most appropriate public health response for disaster victims.
